# Comparison of surgical skill acquisition by UK surgical trainees and Sierra Leonean associate clinicians in a task‐sharing programme

**DOI:** 10.1002/bjs5.50122

**Published:** 2018-12-24

**Authors:** B. Liu, L. M. Hunt, R. J. Lonsdale, H. S. Narula, A. F. Mansaray, I. Bundu, H. A. Bolkan

**Affiliations:** ^1^ Department of Continuing Education, University of Oxford Oxford UK; ^2^ General Surgery Directorate Sheffield Teaching Hospitals NHS Trust Sheffield UK; ^3^ Vascular Surgery Directorate Sheffield Teaching Hospitals NHS Trust Sheffield UK; ^4^ General Surgery Directorate, Chesterfield Royal Hospital NHS Foundation Trust Chesterfield UK; ^5^ CapaCare, Trondheim Norway and Freetown Sierra Leone; ^6^ Institute of Cancer Research and Molecular Medicine, Norwegian University of Science and Technology Trondheim Norway; ^7^ Department of Surgery, St Olav's Hospital, Trondheim University Hospital Trondheim Norway; ^8^ Department of Surgery, Connaught Hospital Freetown Sierra Leone; ^9^ Ministry of Health and Sanitation University of Sierra Leone Freetown Sierra Leone; ^10^ College of Medicine and Allied Health Sciences University of Sierra Leone Freetown Sierra Leone; ^11^ Serabu Mission Hospital Serabu Sierra Leone

## Abstract

**Background:**

Task‐sharing in surgery is well established, with associate clinicians performing successful surgery in many countries. Little is known about the process of surgical skill acquisition by associate clinicians, or whether this differs from that of doctors.

**Methods:**

A blinded experimental study compared surgical skill acquisition by Sierra Leonean associate clinicians enrolled in an essential and emergency surgery training programme with that of a matched group of UK surgical trainees. After identical instruction, practice time and with identities disguised, trainees were videoed performing simulated surgery. Trainees were marked on 12 performance parameters and five behaviour characteristics using validated tools and qualitative comment.

**Results:**

The Sierra Leonean group comprised 19 associate clinicians and one doctor; the UK group comprised 20 doctors in their first 5 years of training. The UK group had significantly more surgical and postgraduate experience than the Sierra Leonean group. The Sierra Leonean trainees outperformed the UK trainees on three of the 12 performance parameters and four of the five behaviour characteristics. UK trainees did not outperform Sierra Leonean trainees on any parameter or characteristic. Qualitative differences in learning style were observed.

**Conclusion:**

Sierra Leonean associate clinicians demonstrated equal or superior skill in all objective parameters tested, despite having less experience than the UK doctors.

## Introduction

Like many countries in sub‐Saharan Africa^1^ ^2^, Sierra Leone suffers from a critical shortage of surgeons, with only ten specialist surgeons in the government hospitals^3^ and 26 in not‐for‐profit non‐governmental hospitals^4^ providing surgical (including obstetric) care for a population of seven million people^5^. The Lancet Commission on Global Surgery^1^ recommends a minimum of 20 surgeons, obstetricians and anaesthetists per 100 000 population. Task‐sharing, defined as the rational redistribution of healthcare workers^6^, may be part of the solution to this shortfall. It is well established in East and Central Africa^7^ ^8^ and to a lesser extent in West Africa^9^, and has been shown to be cost‐effective and efficient^10^, ^11^, ^12^ without compromising surgical outcome^13^ ^14^.

Community health officers in Sierra Leone are a cadre of associate clinicians who have undergone a 3‐year basic medical diploma and work in community health centres and hospitals providing general medical care. A 2‐year surgical task‐sharing programme commenced in Sierra Leone in 2011 to expand the practice of selected community health officers and non‐specialist doctors in providing essential and emergency surgery and obstetric care. For logistical reasons, the opportunity for doctors to enrol in the programme has been limited and, to date, 55 of 57 (96 per cent) of the trainees in this programme are health officers. The 5‐year outcome, productivity and safety of this surgical training programme have been described recently^15^. The training scheme evolved over the first few years, and a designated basic surgical skills course was introduced in 2014.

The primary aim of this study was to evaluate whether, with identical training, those enrolled in this predominantly associate clinician training programme could achieve the same level of competence, in the same time, as matched UK trainees, all of whom were medically qualified. A secondary aim was to understand any qualitative difference in learning between the two groups of trainees.

## Methods

All participants were consenting volunteers, recruited between October 2015 and April 2016. The UK group involved medically qualified UK surgical trainees in their first 5 years of training. The Sierra Leonean group involved surgical trainees (19 associate clinicians and 1 newly qualified doctor) enrolled in the surgical training programme.

Sierra Leonean trainees were invited to volunteer when they attended the base hospital for training or other purposes. Consecutive trainees attending the base hospital were approached, and all agreed to take part. UK trainees were invited to volunteer when they attended regional in‐service training days. All those attending were invited; a few individuals also asked to take part, having heard about the study through colleagues. Consecutive volunteers were recruited with no selection process. Any trainee who had previously performed simulated or actual venous cut‐down was excluded.

Skill acquisition was assessed using a medium‐fidelity simulated venous cut‐down model (Limbs & Things, Bristol, UK). The simulation requires the following surgical skills: incision, dissection, venotomy, knot‐tying and suturing. This construct was selected for ease of video assessment, speed of assessment and unfamiliarity to all trainees. It had the advantage of being an attractive skill for both groups, being part of life‐saving skills for obstetrics necessary in Sierra Leone and of Advanced Trauma Life Support training in the UK.

Both groups were exposed to identical training materials over a 1‐h period, consisting of an instructional video, a paper fact sheet, and access to the simulation model to practise as often as they wished. All materials were in English. English is the official language and language of higher education in Sierra Leone, although the second or third language for participants in the Sierra Leonean group. To mitigate this possible bias, the operator in the training video was a Sierra Leonean surgeon speaking in English. There was additional instructional commentary on the training video by one of the authors familiar to both groups of trainees. After 1 h, trainees each performed the simulation with one assistant taken from the same study group. Each assistant was only used once. To mask ethnic identity, all participants wore identical surgical gowns and opaque gloves, and videos showed only the covered arms and hands. Work surfaces were covered with identical drapes. Procedures were video‐recorded. Participants were asked to talk about what they were doing during the video, and all speech was removed and dubbed back on by a single investigator.

Videos were randomized and uploaded online. They were viewed in unedited format by two consultant surgeons independently. Both assessors participate in UK National Selection at Speciality Trainee (general and vascular surgery) Year 3 level and were therefore familiar with the scoring methods used. Performance parameters were assessed using validated Objective Structured Assessment of Technical Skills (OSATS), and trainee behaviour characteristics were assessed using a 5‐point Likert scale. Each participant completed a 12‐question true/false multiple choice question (MCQ) test to ascertain factual knowledge about the procedure and its application.

Ethical approval for the study was obtained from the University of Oxford in the UK; an ethical committee waiver was granted in Sierra Leone as no patients were involved in the study.

### Statistical analysis

Assuming considerable intragroup variation, 20 participants in each group had a power of 0·8 to detect a 10 per cent difference between groups. The null hypothesis assumed no difference in performance and skills acquisition between the two groups. Categorical data were analysed with the χ^2^ test, parametric data with Student's *t* test, and non‐parametric data with two‐tailed Mann–Whitney *U* tests using a 95 per cent confidence level. Spearman's rank correlation test and a logistic regression model were used to identify independent factors that might predict candidates' performances using Microsoft Excel® (Microsoft, Richmond, Washington, USA) and SPSS® version 22 software (IBM, Armonk, New York, USA).

Qualitative analysis was undertaken using the quasi‐quantitative methods described by Burnard^16^. This analysis was used to code words and phrases within assessors' comments. Repetition, emphasis, contradictions and words associated with learner characteristics were categorized according to Miller's method^17^ into: cognitive patterns; behavioural patterns; and adherence to instructions.

## Results

More experienced UK trainees volunteered, with the unintended consequence that the UK group had spent significantly more time in surgical training than the Sierra Leonean group (mean 3·04 *versus* 1·78 years respectively). The Sierra Leonean trainees were significantly older and less likely to have undertaken a basic surgical skills course than the UK trainees (*Table* 1).

**Table 1:** Description of participants

	UK trainees (*n* = 20)	Sierra Leone trainees (*n* = 20)	*P* ‡
Age (years)*	27·25	33·95	< 0·001
Sex ratio (M : F)	12 : 8	17 : 3	0·076§
Surgical experience (years)*, †	3·04	1·78	0·017
Completed formal basic surgical skills course (%)	16	10	0·046§

* Values are mean.

† Time from first entry into surgical training to day of experiment.

‡ Student's *t* test, except

§ χ^2^ test.

No difference in performance was seen between the two groups for the MCQ test, nine of the 12 performance parameters and the assessors' global impression. The Sierra Leonean trainees outperformed the UK trainees on three of the 12 performance parameters and four of the five trainee behaviour characteristics. UK trainees did not outperform the Sierra Leonean trainees on any parameter or characteristic (*Table* 2).

**Table 2:** Performance parameters and behaviour characteristics

	UK trainees (*n* = 20)	Sierra Leone trainees (*n* = 20)	*P* ‡
Mean MCQ test score (maximum 12)	8·0	8·9	0·295§
Time to complete simulation (s)	456	522	0·149§
Performance parameters of simulated surgery*			
Respect for tissue	3	3	0·238
Time and motion	3	3	0·960
Instrument handling	3	3	0·872
Choice of instruments + suture	3	4	0·005
Incision and venotomy	2	3	0·589
Insertion of cannula	3	3	0·362
Knot‐tying	3	3	0·631
Errors	2	3	0·016
Flow of procedure	3	3	0·802
Use of assistant	2	2·5	0·001
Knowledge of the procedure	3	3	0·123
Global impression	2	3	0·183
Behaviour characteristics†			
Appropriate confidence	2	3	0·053
Meticulousness	2	3	0·029
Communication (with assistant)	2	3	0·006
Verbalizes steps	2	3	< 0·001
Assessors' estimate of trainees' previous experience	2	3	0·490

* Median 5‐point Objective Structured Assessment of Technical Skills (OSATS);

† median 5‐point Likert scale. MCQ, multiple choice question.

‡ Mann–Whitney *U* test, except

§ χ^2^ test.

Assessors were asked to make an estimate of the length of time the trainees had been in training based on the video performance. The correlation analysis revealed that years of surgical experience appeared to have a greater influence on the performance of UK trainees (*Table* 3). Regression analysis indicated that being a Sierra Leonean trainee (*P* = 0·001), older age (*P* = 0·011) and having more actual surgical experience (*P* = 0·004) all predicted a higher estimation of length of time in surgical training. Only being a Sierra Leonean trainee independently predicted a higher Global Impression Score (*P* = 0·006) (*Table* 4).

**Table 3:** Correlation between assessors' estimate of trainees' surgical experience *versus* actual experience

	UK trainees	Sierra Leone trainees
Correlation coefficient (*R*)	0·52	−0·09
*P*	0·017	0·697

**Table 4:** Ordinal logistic regression: independent factors affecting the Global Impression Score and assessors' estimated years of experience

	Wald χ^2^	d.f.	*P*
Factors affecting Global Impression Score			
Completed basic surgical skills course	2·37	1	0·124
MCQ test score	13·04	7	0·071
Trainee type	7·63	1	0·006
Age	1·86	1	0·173
Experience	1·48	1	0·224
Factors affecting estimated years of experience			
Completed basic surgical skills course	1·76	1	0·185
MCQ test score	9·25	7	0·236
Trainee type	10·41	1	0·001
Age	6·52	1	0·011
Experience	8·36	1	0·004

MCQ, multiple choice question.

Results and examples of the qualitative assessments for types of comment are shown in *Table* 5. Assessors made more negatively coded critiques for both groups. Procedures performed to expectation often did not attract comment. Although there were more negative comments for the UK group (45 *versus* 26 respectively), there were no significant differences between the two groups (*P* = 0·072). Positive critiques in both groups and in all domains were generic. Negative comments were more revealing as ‘dangerous technique’ and ‘risk of needlestick injury to the assistant’ would require direct intervention from trainers in a real‐life procedure. Sierra Leonean trainees showed a preference for verbal learning, talking through the steps of the procedure and their cognitive processes. They interacted more with their assistant and were bolder in their actions.

**Table 5:** Qualitative analyses of comments

	UK		Sierra Leone	
	Positive comments	Negative comments	Positive comments	Negative comments
Adherence to instructions				
No. of comments	2	14	4	5
Examples	Good length of skin incision; good length of vein exposed	Did not close skin; left the stylet in; venotomy performed without ligatures	Good knowledge of procedure	Venotomy and cannulation not shown; incision in the wrong place
Cognitive patterns				
No. of comments	4	14	4	10
Examples	Good communication; I actually would have done the same as him/her; purposeful dissection	Hesitant; silent; risk of needlestick injury to the assistant; uncertain whether to cut the stay suture	Intuitive; verbalizes well; recognized that initial venotomy was inadequate	Panicked: ‘Oh it's bleeding'; rushed
Behavioural patterns				
No. of comments	5	17	4	11
Examples	Careful dissection; good knot; good skin incision	Clumsy; sloppy; dangerous technique; air knots; cut the vein with skin incision	Good dissection; fluid movement; careful and good technique	Rough; does not run knot down well; complex suturing to the cannula

## Discussion

Despite evidence of safety^13^, ^14^, ^15^, task‐sharing is still met with scepticism by some. Almost 80 per cent of surveyed UK patients would rather wait longer to be operated on by a doctor than have the procedure done sooner by a non‐medically qualified surgeon^18^. Associate clinicians are crucial for improving patient care and access to surgical service delivery in Sierra Leone. Failure to recognize their abilities is a barrier to their successful deployment and retention^15^.

It is unusual for medically qualified and non‐medically qualified surgical trainees to receive identical training, and therefore difficult to make comparisons regarding skill acquisition. The present study found that, with identical training, Sierra Leonean community health officers showed equal or superior skill acquisition, greater confidence and better communication than UK surgical trainees, despite UK trainees having more previous surgical experience.

Categorization of behaviour patterns can help an understanding of how individuals learn^19^. The VAK/VARK (Visual, Aural, Read/Write and Kinesthetic) model^20^ proposes visual, auditory, verbal and tactile learning modalities. Gardner^21^ expanded this to include logical, social and solitary dimensions to learning. The patterns seen in the present study suggest the Sierra Leonean trainees had a preference for verbal learning. They were excellent at verbalizing the steps of the procedure and more vocal in expressing their cognitive processes than the UK trainees. They were bolder in their movements (tactile learning), which may have appeared as ‘rushed’ but also as more confident. They showed social learning with more communication and use of the assistant. They were logical with instrument selection and more likely to identify their errors, understanding when the procedure was not progressing as expected with a greater degree of anticipation than the UK trainees. These qualities may have led to their better overall performance.

Previous years of surgical experience correlated with performance and the assessors' estimate of length of time in surgical training amongst the UK trainees. Previous experience and transferable skills might be expected to improve effectiveness when learning a new procedure. This was not seen amongst the Sierra Leonean trainees, who demonstrated equal poise to perform the procedure regardless of experience. It appeared they either had more trust in the training materials or paid more attention to them. This is consistent with the assessors' findings that the Sierra Leonean trainees demonstrated greater confidence.

The demonstrator in the instructional video was Sierra Leonean, and his features were not masked. This may have led to a role‐modelling effect on the trainees' learning. Medical students have been shown to identify their ideal clinical role model along racial grounds^22^. Further assessment of the role‐modelling effect may have implications for training programmes in low‐resource settings using expatriate trainers. As interest in global surgery burgeons, this could also be helpful to individual trainers and colleges.

Detractors could argue the Sierra Leonean trainees' performance simply reflects more didactic learning rather than being a true representation of cognitive process. Perhaps the Sierra Leonean trainees simply followed the instructions better. The observations suggest, however, that the Sierra Leonean trainees adopted a more conscious and stepwise approach to completing the task, with deliberation and verbalization of what they aimed to achieve for each step of the procedure, voicing potential pitfalls of the steps before they interacted with the simulation, recognizing and correcting errors if necessary. This transformed them from being technicians to deliberating practitioners with contextual awareness, a hallmark of deliberate practice^23^.

In contrast the UK trainees were more silent, showed less preparation, and appeared less discriminatory in their choice of instruments and sutures. This could indicate lower cognitive engagement with the task, perhaps being accustomed to an environment where they are handed equipment without the need to consider its suitability. Perhaps UK trainees may simply have been less talkative with their cognitive processes occurring silently. This could make them appear more hesitant. The cognitive process seems more explicit in the more communicative Sierra Leonean trainees.

This study aimed to see whether, given the same training, Sierra Leonean associate clinicians achieved the same level of competence as medically qualified UK trainees. Superiority had not been anticipated, raising a number of questions. It may reflect cultural background. Sierra Leoneans tend to be vocal. Perhaps this can contribute to their ability to learn surgical techniques. Training in low‐resource settings can be hard to obtain and is seen as a privilege; this could contribute to an eagerness to learn. In addition, the vocal commentary of the Sierra Leoneans often alluded to recognition of their crucial role as the only professionals able to perform this task to save lives in deprived localities. The poorer performance of the UK trainees, despite their greater experience and in many cases more formal training, could represent complacency among those who benefit from a sophisticated training structure, or a knowledge that they are exceptionally unlikely to be left alone with this task in the near future.

There are limitations to this study, including assessments of more complex procedures, real‐life surgery and a closer examination of cultural differences in the cognitive process of learning. Nevertheless, the study provides some insight into the educational ability of associate clinicians performing surgery in a low‐resource setting. It is reassuring that they were not found lacking. The poorer performance of the UK trainees raises questions about whether we currently provide early‐years trainees with the best possible training environment. Perhaps they could learn from those facing much tougher working environments early in their careers.
